# Biomechanical analysis of spinal range of motion and intervertebral disc loadings in normal and adolescent idiopathic scoliosis models

**DOI:** 10.3389/fbioe.2025.1473776

**Published:** 2025-02-19

**Authors:** Haikuan Wang, Zhengwei Ma, Zhihua Wu, Yuanfang Lin, Jie Yu, Xin Qian, Sili Jian, Yueli Sun, Wei Wei, Xiang Yu, Ziyang Liang

**Affiliations:** ^1^ South China Research Center for Acupuncture and Moxibustion, Medical College of Acu - Moxi and Rehabilitation, Guangzhou University of Chinese Medicine, Guangzhou, China; ^2^ College of Urban Transportation and Logistics, Shenzhen Technology University, Shenzhen, Guangdong, China; ^3^ The First Affiliated Hospital of Guangzhou University of Chinese Medicine, Guangdong Clinical Research Academy of Chinese Medicine, Guangzhou, China; ^4^ Department of Tuina and Spinal Orthopaedics in Chinese Medicine, Shenzhen Traditional Chinese Medicine Hospital, The Fourth Clinical Medical College of Guangzhou University of Chinese Medicine, Shenzhen, Guangdong, China; ^5^ Key Laboratory of the Ministry of Education of Chronic Musculoskeletal Disease, Longhua Hospital, Spine Institute, Shanghai University of Traditional Chinese Medicine, Shanghai, China; ^6^ Laboratoire de Biomécanique Appliquée (UMRT24), Aix-Marseille Université/Université Gustave Eiffel, Marseille, France; ^7^ Department of Orthopedics, The Second Xiangya Hospital of Central South University, Changsha, China

**Keywords:** adolescent idiopathic scoliosis, Lenke type, biomechanics, finite element, intervertebral disc, range of motion, therapeutic exercises

## Abstract

**Objective:**

While the Lenke classification enhances our structural understanding of adolescent idiopathic scoliosis (AIS), the biomechanical implications for spinal range of motion (ROM) and intervertebral disc (IVD) loadings remain unclear. This study aims to quantitatively explore and compare these biomechanical responses in normal thoracolumbar spines and those with various curvatures of Lenke types under pure bending conditions.

**Methods:**

The baseline thoracolumbar finite element (FE) model was derived from a comprehensive human body FE model, validated, and calibrated against spinal responses under dynamic compression and quasi-static bending conditions. Using mesh morphing, AIS models of Lenke 1, Lenke 2, Lenke 3, and Lenke 5 were established to represent their respective spinal curvatures. Pure bending moments of ±7.5 Nm in flexion-extension, lateral bending, and axial rotation were applied to both normal and AIS models. Global spinal ROM and ROM of spinal segments T1-T6, T7-T12, and L1-Sacrum were measured under each loading condition. IVD mechanical loadings, including force, moment, and VonMises stress, were also evaluated and compared across all models.

**Results:**

AIS models showed higher principal ROM compared to the normal model, with Lenke 2 having the highest ROM from T1-Sacrum and Lenke 3 the highest ROM from T6-12. AIS models exhibited more asymmetry in segmental ROM, particularly in the lumbar spine during lateral bending and axial rotation. IVD mechanical loadings varied significantly between normal and AIS models, influenced by spinal curvature types. AIS models had higher secondary moments and shear forces, especially under flexion-extension. The highest stress was mostly observed in the frontal IVD regions under flexion which was greatly reduced under extension. Lateral bending caused the highest stress predominantly on the same side as the loading direction in the IVD regions. The IVDs of T6-T7 and T12-L1 showed even stress distribution under axial rotation, while the right IVD regions of L5-Sacrum sustained the highest stress under right axial rotation, and the left regions under left axial rotation. In Lenke 3 and Lenke 5 models, the right (concave) regions of the T12-L1 IVD consistently sustained higher stress levels, regardless of the loading conditions applied.

**Conclusion:**

This study underscores significant biomechanical differences between normal and AIS models, revealing intricate interactions within scoliotic spines and enhancing our understanding of AIS biomechanics. These insights can aid in better diagnosis, treatment planning, and prognosis. Extension-focused therapeutic exercises may reduce stress on anterior IVDs, potentially lowering the risk of low back pain or disc herniation, while careful management of rotational exercises can help minimize stress in the lower lumbar regions.

## 1 Introduction

Adolescent idiopathic scoliosis (AIS) is a complex three-dimensional deformity of the spine, with a Cobb’s angle greater than 10° when measured in the coronal plane (Miller et al., 2012). The prevalence of AIS varies worldwide, ranging from 0.93% to 12%, making it a significant public health concern (Sung et al., 2021). In the United States alone, scoliosis patients make more than 600,000 visits to private physician offices annually, with approximately 30,000 children being fitted with braces and 38,000 patients undergoing spinal fusion surgery (National Scoliosis Foundation, 2024; Kumar et al., 2024).

To facilitate effective diagnosis, treatment planning, and prognosis, various classification systems have been developed to categorize AIS based on curve patterns, severity, and other factors. Among the most widely recognized and utilized is the Lenke classification system, introduced in 2001 (Lenke et al., 2001). This system has become the gold standard for categorizing AIS curve patterns due to its comprehensive nature and enhanced reliability (Lenke et al., 2001). The Lenke classification identifies six main curve types (Lenke 1–6). The most common type, Lenke 1 (main thoracic curve), occurs in 45%–51% of AIS patients, followed by Lenke 3 (double major curve) at 20%–22%, Lenke 5 (main thoracolumbar/lumbar curve) at 15%–20%, and Lenke 2 (double thoracic curve) at 10%–15% (Lehman et al., 2008; Tavares et al., 2017; Xu et al., 2015). While the Lenke classification has significantly improved our understanding of AIS from a structural perspective, the biomechanical implications of these different curve types remain incompletely understood.

Spinal range of motion (ROM) is a critical aspect of spinal biomechanics that is likely to be affected by the various curve patterns in AIS. This factor may not only influence the progression of the deformity but also play a crucial role in the development of pain, functional limitations, and long-term complications associated with AIS (Hartley et al., 2022; Mehkri et al., 2021). Galvis et al. (2016) measured the spinal mobility of patients with right thoracic AIS, with apices at T6-T10, under their maximum bent position in the sagittal and coronal planes. Contrary to expectations, these AIS patients did not exhibit reduced mobility compared to the normal population but demonstrated greater mobility, particularly in segments directly above and below the curve apex (Galvis et al., 2016). Similarly, Eyvazov et al. (2017) measured thoracolumbar spinal ROM in AIS patients with Lenke 5 curves in the coronal, sagittal, and axial planes. Their findings suggest that spinal ROM is affected by AIS curve magnitude, with significantly lower mobility observed in more severe spinal curves (>40°). Although these studies have demonstrated that AIS patients generally exhibit altered spinal kinematics compared to healthy individuals, there is a lack of consistent findings. This inconsistency may be due to treating AIS as a homogeneous group without considering the potential differences among Lenke types.

The three-dimensional spinal deformity caused by AIS has been widely reported to result in asymmetric loadings of intervertebral discs (IVD) (Skalli and Vergari, 2018). Despite the lack of experimental data on IVD mechanical loadings in AIS patients, most studies on this topic rely on finite element (FE) modeling. Zhang et al. (2021) developed a patient-specific lumbar spine FE model and compared the IVD stress distribution with that of a normal model under flexion-extension, lateral bending, and axial rotation. Their findings suggest that IVD stress distribution patterns differ between the concave and convex sides of the scoliotic curve. Similarly, Kamal et al. (2019) created a thoracolumbar spine FE model incorporating trunk and muscle forces based on a specific AIS patient to simulate stress-modulated growth in AIS, using theories like Hueter-Volkmann to predict curve progression and vertebral wedging over time. They suggested that reaction moments in the scoliotic spine amplified IVD stresses on the posteroconcave side. D’Andrea et al. (2023) developed patient-specific FE models of the spine, ribcage, and pelvis for five AIS patients (four patients with Lenke 1 and one patient with Lenke 2) using a morphing technique based on a normative template FE model. They evaluated the principal stress in the IVDs of the apical vertebral level and found that the anterior IVD regions sustained higher stress than the posterior regions initially, regardless of the convex or concave side. However, during follow-up periods under the loading of gravity force, the concave side consistently sustained higher stress levels than the convex side. While these studies have provided important insights into IVD loadings among AIS patients, the models developed often represent a single patient (Zhang et al., 2021; Kamal et al., 2019) or a small cohort (D’Andrea et al., 2023), limiting the generalizability of results. Additionally, these studies focus often on static loading conditions (Kamal et al., 2019; D’Andrea et al., 2023), which may not fully capture the complex dynamic loads experienced by the spine during daily activities.

Therefore, in this article, we aim to explore the biomechanical behavior of the spine, focusing on spinal ROM and IVD mechanical loadings in individuals with AIS and those without scoliosis, using FE modeling. Our study encompasses a control model without scoliosis and includes models representing Lenke 1, Lenke 2, Lenke 3, and Lenke 5 curvatures. By analyzing these different models, we aim to provide a comprehensive understanding of the biomechanical differences between the normal spine and various Lenke types, thereby contributing to more effective diagnosis, treatment planning, and prognosis for AIS patients.

## 2 Materials and methods

### 2.1 Thoracolumbar spine FE model

**FIGURE 1:**
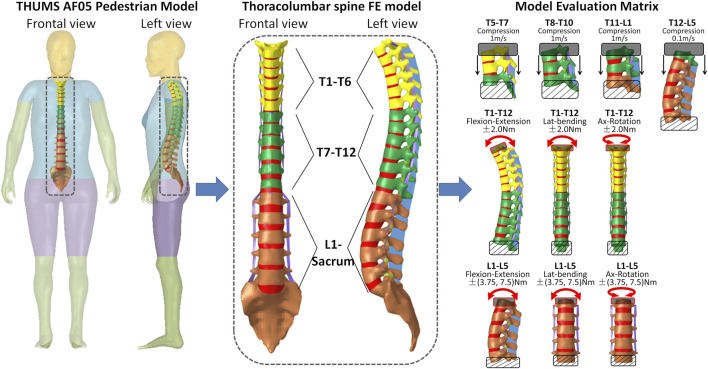
FE model of thoracolumbar spine extracted from the THUMS AF05 pedestrian model and the model biomechanical evaluation matrix.

The FE model of the thoracic and lumbar spinal segments (presented in Figure 1) was isolated from the small-sized female pedestrian model (154 cm height and 52 kg weight) featured in the fourth version of the Total Human Model for Safety (THUMS AF05 pedestrian model). This model was selected due to its close alignment with the anthropometry of adolescents, who are more commonly affected by AIS. Research shows that female adolescents aged 12 to 14 are up to 8.4 times more likely to develop AIS than their male counterparts (Daniel et al., 2020). According to clinical growth charts from the National Center for Health Statistics (https://www.cdc.gov/growthcharts/clinical_charts.htm), the median height and weight for girls in this age range fall between 154.4 cm and 161.3 cm, and 52.2 kg and 59.7 kg, respectively. Additionally, the model’s posture closely resembles a neutral standing position, aligning with the pure bending conditions of our study, which began from a stress/strain-free initial state. In contrast, occupant models feature a more curved spine to mimic seated postures with pre-existing stress/strain. These factors make the THUMS AF05 pedestrian model more suitable for simulating AIS biomechanics in this study.

The THUMS AF05 pedestrian model was collaboratively developed by TOYOTA MOTOR CORPORATION and TOYOTA CENTRAL R&D LABS to replicate human body kinematics and internal organ injury responses during pedestrian-car collisions (Watanabe et al., 2011). It was constructed based on high-resolution CT scans of a 38-year-old female with a stature of 154 cm, body mass of 52 kg, and implemented using assumed constitutive material properties. The thoracolumbar spine FE model, extracted from the THUMS AF05 Pedestrian model, comprises a total of approximately 132,123 elements. The model incorporates anatomical structures such as vertebrae, IVDs, ligaments. Cortical bone of the vertebrae is represented by shell elements, employing an elasto-visco-plastic material model with pre-rupture damage effects based on an effective plastic-strain criterion (material parameters detailed in Table 1). The spongy bone of the vertebrae is modeled using solid elements, employing an elastic viscoplastic material model that integrates continuum damage mechanics (material parameters detailed in Table 1). The nucleus of the IVDs is simulated with solid elements and an isotropic elastic plastic material model. The annulus fibrosus is represented using solid elements that mimic the matrix of isotropic highly compressible Fu Chang foam, supplemented by seatbelt elements possessing linear mechanical properties to function as reinforced fibers. The ligaments are simulated with shell elements and a simplified linear elastic material model.

**TABLE 1:** Mechanical properties of the vertebral spongy and cortical bones defined in the original and calibrated thoracolumbar spine FE model.

Material properties	Spongy bone		Cortical bone	
	Original	Calibrated	Original	Calibrated
Density (e−3 g/mm^3^)	1.0	1.0	2.0	2.0
Young’s modulus (MPa)	40.0	40.0	13020.0	13020.0
Poisson’s ratio	0.45	0.25^*^	0.30	0.30
Yield stress (MPa)	1.8	1.8	80.0	80.0
Tangent modulus (MPa)	13.3	13.3	0.0	0.0
Failure plastic strain	0.0	0.06^*^	0.0	0.071^*^
Strain rate coefficients	r_d_ = 1.0, d_c_ = 0.5	r_d_ = 1.0, d_c_ = 0.5	c = 360.7, p = 4.605	c = 360.7, p = 4.605

*The corresponding material parameters were adjusted for the purpose of model calibration, aligning with data found in the literature (Wagnac et al., 2012).

To ensure its biofidelity in simulating pedestrian-car crashes, the biomechanical responses of the THUMS AF05 Pedestrian model have been validated across various body regions (e.g., head, neck, torso, and extremities) and at the full-body level, against data from human cadaveric experiments involving diverse automobile impact scenarios (Watanabe et al., 2011; Iwamoto et al., 2015). However, the biofidelity of the thoracolumbar spine model specifically has not been assessed. Therefore, it was essential to evaluate its biomechanical responses before employing the thoracolumbar spine FE model to investigate the biomechanics of various AIS types in this study. The biomechanical assessment of the thoracolumbar spine FE model was thus conducted on dynamic compression and quasi-static bending conditions (as shown in Figure 1). Initially, the model with its original spinal material properties exhibited responses that fell outside the experimental validation corridors in certain cases. To address this, adjustments were made to the material properties of the vertebral components (outlined in Table 1) to bring the model into alignment with the experimental data. For clarity, the detailed calibration process is provided in the Supplementary Appendix, ensuring the main manuscript remains concise.

### 2.2 AIS thoracolumbar spine FE models

**FIGURE 2:**
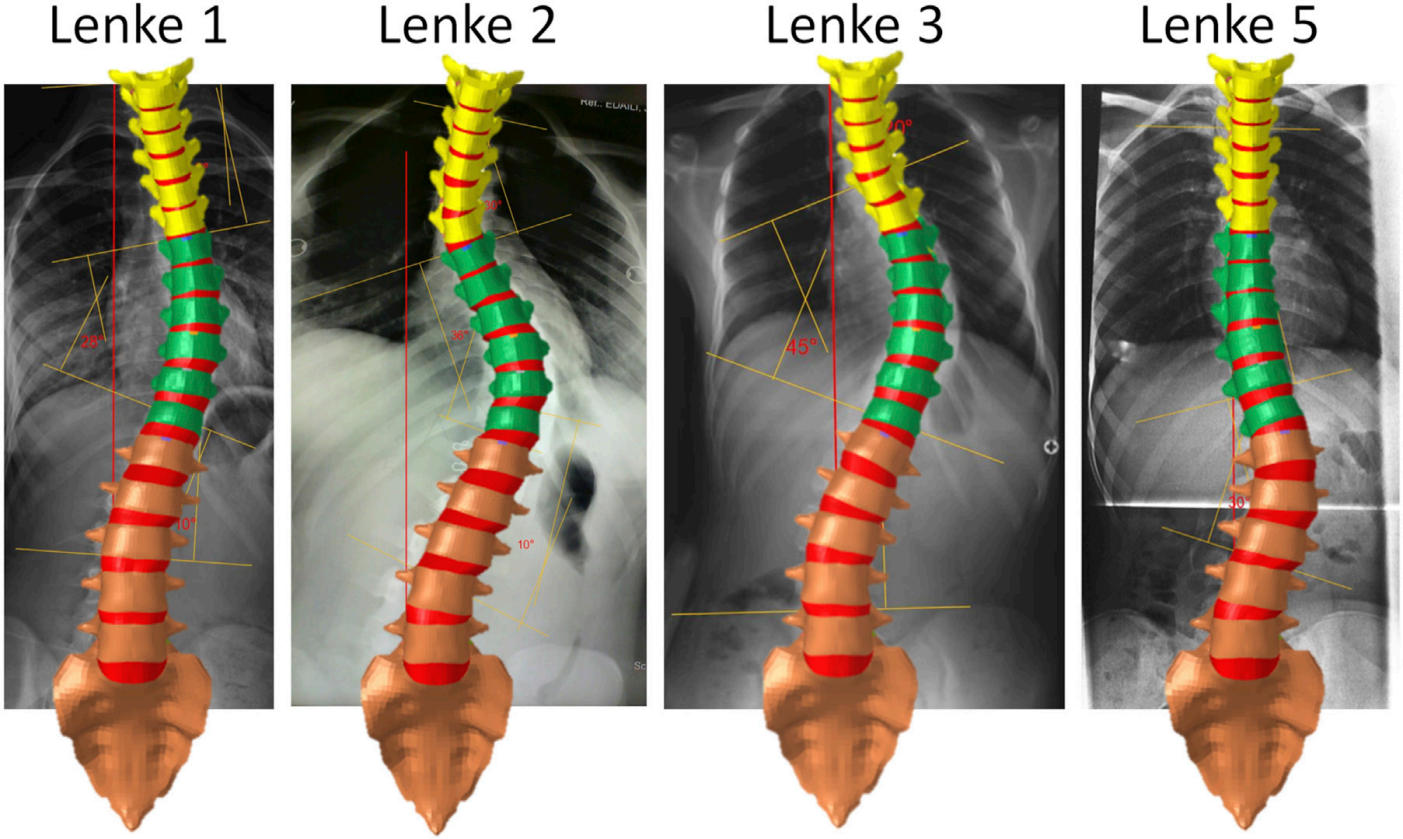
Thoracolumbar spine FE models representing AIS Lenke 1, Lenke 2, Lenke 3, and Lenke 5: coronal spinal curvatures aligned with chosen medical images.

The calibrated thoracolumbar spine FE model was further adapted to simulate various types of AIS, including Lenke 1, Lenke 2, Lenke 3, and Lenke 5. Representative X-ray images corresponding to each AIS type were selected from an open-source dataset (Fraiwan et al., 2022) due to the unavailability of hospital-based medical images. These images were chosen to align with the specific characteristics of each scoliosis type: for Lenke 1, significant coronal curvature in the thoracic spine (T7-T12 coronal Cobb angle of 28°); for Lenke 2, dual coronal curvatures in the thoracic spine (T2-T6 Cobb angle of 30° and T7-T12 Cobb angle of 36°); for Lenke 3, dual major coronal curvatures (T5-T12 Cobb angle of 45° and L1-L4 Cobb angle of 25°); and for Lenke 5, primary coronal curvature in the thoracolumbar or lumbar spine (T12-L3 Cobb angle of 30°), as depicted in Figure 2. Although thoracic scoliotic curves are typically right convex due to anatomical asymmetries, a left-sided scoliosis curve was selected in this study to maintain consistency in convex direction and because superior imaging quality was available for certain subjects with a left convex thoracic curve. Due to the open-source dataset containing only coronal plane images, the same sagittal spinal curvature from the THUMS AF05 pedestrian model (12°, 14°, and 40° for the T1-T6, T7-T12, and L1-L5 sagittal Cobb angles, respectively) was maintained across the healthy model and all Lenke subtypes. Although sagittal curvature was not varied for different Lenke types, the focus on coronal plane alterations in this study aligns with clinical practice and should provide valuable insights into the biomechanical differences across Lenke subtypes.

To accurately replicate the spinal coronal curvatures corresponding to each Lenke subtype, the IVDs within the calibrated thoracolumbar spine FE model were systematically deformed by iteratively adjusting the collateral disc heights using a mesh morphing technique. This deformation process was facilitated through the utilization of manual freehand morphing and mapping functions available in HyperMesh (version 2021, Altair Engineering, Troy, MI). The objective was to iteratively manipulate the IVDs until the coronal curvatures of the model aligned closely with the curvatures observed in the selected medical images (as illustrated in Figure 2). For this manipulation, specific nodes located on the upper surface of each IVD were designated as moving nodes, while nodes on the lower surface of each IVD were designated as fixed nodes. The moving nodes associated with the upper vertebrae were allowed to move in all degrees of freedom, with user-defined displacements and rotations, while the fixed nodes corresponding to the lower vertebrae remained unchanged. This iterative process was repeated for each IVD within the thoracolumbar spine FE model, ensuring that the resulting coronal spinal curvatures harmonized with those displayed in the chosen medical images (as depicted in Figure 2).

### 2.3 Biomechanical analysis under pure bending moments

**FIGURE 3:**
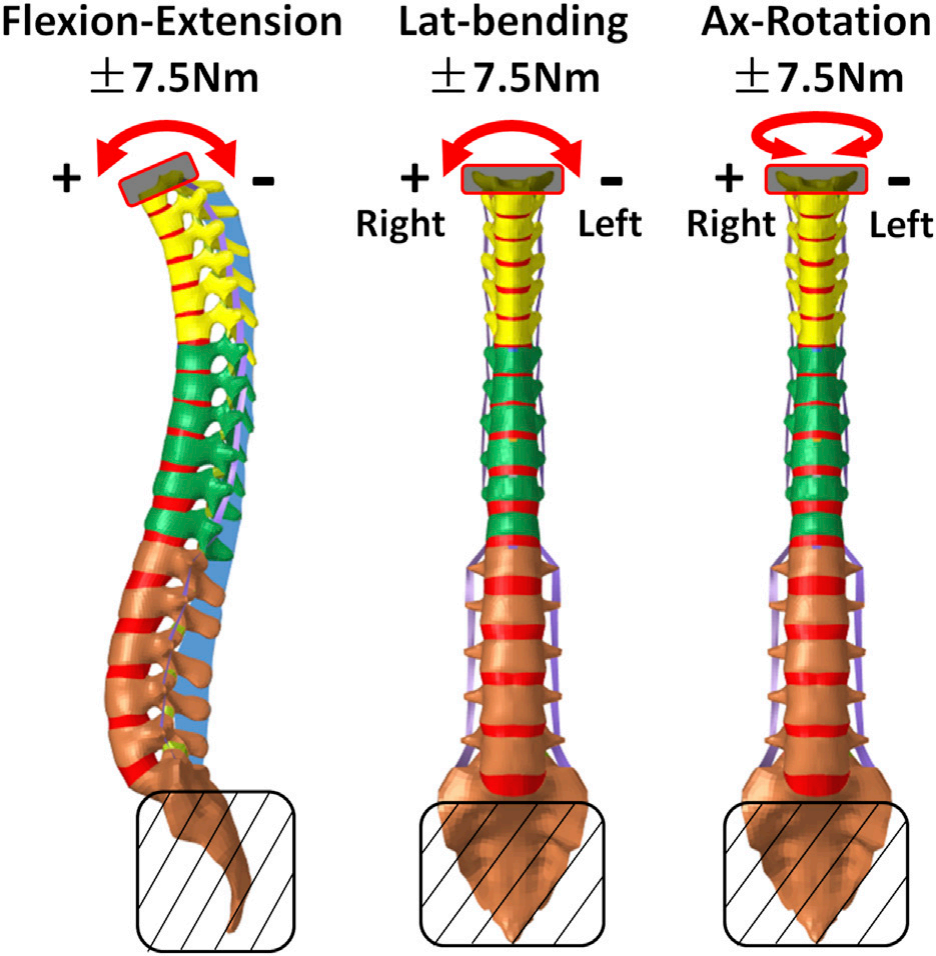
Schematic boundary conditions for biomechanical analysis of normal and AIS (Lenke 1, Lenke 2, Lenke 3, and Lenke 5) thoracolumbar spine models.

Biomechanical analysis was ultimately conducted on both the normal and AIS (Lenke 1, Lenke 2, Lenke 3, and Lenke 5) thoracolumbar spine FE models under the influence of pure bending moments (as illustrated in Figure 3). The sacrum was constrained across all degrees of freedom, while the upper portion of T1 was subjected to pure moments of ±7.5 Nm, aligning with flexion-extension, lateral bending, and axial rotation directions. These applied pure moments were consistent with those employed in previous experimental studies (Rohlmann et al., 2001). ROM measurements were assessed between T1-T6, T7-T12, and L1-Sacrum to capture spinal flexibility across key segments under each bending condition for flexion-extension, lateral bending, and axial rotation. The selection of T6, T12, and the sacrum is crucial for several reasons. T6 and T12 are located near transition points in the spine where the curvature often changes direction, making them relevant landmarks for assessing Cobb angles, particularly in Lenke 1, 2, and 3 subtypes (see Figure 2), where thoracic curvature is prominent. Furthermore, T6 divides the upper and lower thoracic spine, while T12 marks the transition from the thoracic to the lumbar spine, both of which are important regions for evaluating scoliosis-related deformities. The sacrum, being the final vertebra, serves as a biomechanical anchor for the spine and pelvis, and its stability is vital for understanding overall spinal mechanics. These anatomical landmarks also help capture variations in curvature and loading patterns in both the coronal and sagittal planes. Additionally, the load distribution on the IVDs was also evaluated for each model and pure bending condition. The biomechanical responses of the AIS models were compared to those of the normal thoracolumbar spine model to assess the relationship between various spinal curvatures and spine flexibility, as well as IVD loadings, in individuals with and without AIS.

All FE simulations in this study were performed with the explicit solver in LS-DYNA 971 R11.1 (LSTC. Livermore, CA, United States) on an Intel Xeon (2.20 GHz) workstation with 24 processors.

## 3 Results

### 3.1 Spinal range of motion analysis

**FIGURE 4:**
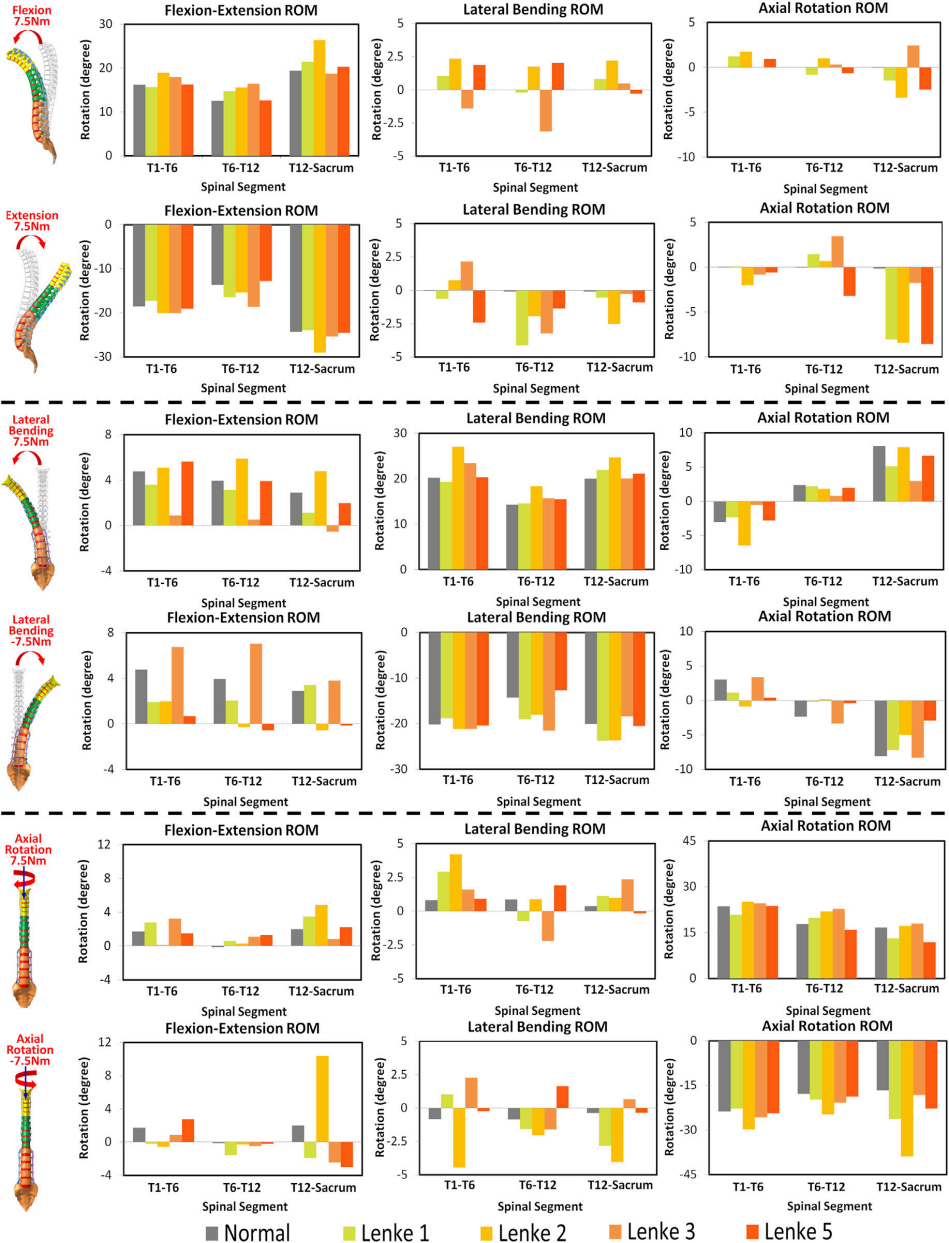
Three-dimensional ROM of spinal segments T1-T6, T6-T12 and T12-Sacrum under flexion-extension, lateral bending and axial rotation in normal and AIS (Lenke 1, Lenke 2, Lenke 3, and Lenke 5) thoracolumbar spine models.

The constructed AIS models, exhibiting primary left-sided scoliosis with curvatures ranging from 28° to 45°, showed notable differences from the normal spine in segmental ROM and coupled motion patterns (Figure 4). The final thoracolumbar spinal curvatures under the applied loadings are shown in Figure 3A of the Supplementary Appendix. Despite these variations, a consistent trend was always observed: the AIS models consistently exhibited higher ROM at the loading direction throughout the thoracolumbar spine segment (hereafter referred as T1-Sacrum) under the moments of flexion-extension, lateral bending and axial rotation, compared to the normal model. Among the AIS models, the ROM was consistently highest in Lenke 2, followed by Lenke 3, Lenke 1, and finally Lenke 5, as outlined in Table 2. In the spinal segment T6-12, which exhibits the greatest deformity angle in Lenke 1, Lenke 2, and Lenke 3 models, the ROM at the loading direction consistently surpassed that of the normal model, with Lenke 3 displaying the highest ROM, followed by Lenke 2 and Lenke 1 (refer to Table 2).

**TABLE 2:** ROM of T6-T12 and thoracolumbar spine segment (T1-Sacrum) at the loading direction under flexion-extension, lateral bending and axial rotation moments of 7.5Nm: comparison between the normal model and different AIS models.

ROM (°) Loading	Normal		Lenke 1		Lenke 2		Lenke 3		Lenke 5	
	T1-Sacrum	T6-T12	T1-Sacrum	T6-T12	T1-Sacrum	T6-T12	T1-Sacrum	T6-T12	T1-Sacrum	T6-T12
Flexion-Extension	104.7°	26.2	109.5	31.2	125.4	31.0	117.3	35.0	105.6	25.4
Lateral bending	109.1°	28.6	117.5	33.7	132.9	36.4	120.3	37.2	110.7	28.2
Axial Rotation	116.2°	35.6	122.5	39.6	157.7	46.7	130.2	43.5	117.5	34.7

Under flexion-extension loading, the normal spine’s ROM in the T1-T6 and T12-Sacrum segments was 84.4% and 79.0% of Lenke 2, respectively, and 74.7% of Lenke 3 in T6-T12. AIS models also showed substantial coupled motions, with lateral bending ranging from 4.1° to 10.0° and axial rotation from 2.8° to 12.3°, compared to minimal coupled motion in the normal spine (<0.2°). In the lumbar region (T12-Sacrum), the AIS models had the highest coupled axial rotation (4.2°–8.6°), with Lenke 2 showing the greatest lateral bending.

Under lateral bending and axial rotation loadings, both the normal spine and AIS models exhibited significant coupled motions. The normal spine showed comparable coupled flexion-extension ROM to the AIS models (11.6° vs 7.9°–17.6°), but had higher coupled axial rotation during lateral bending (14.8° vs 8.7°–11.6°). Under axial rotation loadings, the Lenke 2 model demonstrated a coupled lateral bending ROM of 16.6°, which was four times higher than the normal model and 2.5 times greater than that of the Lenke 1 model, the second highest among AIS models.

### 3.2 Intervertebral disc mechanical loading analysis

**FIGURE 5:**
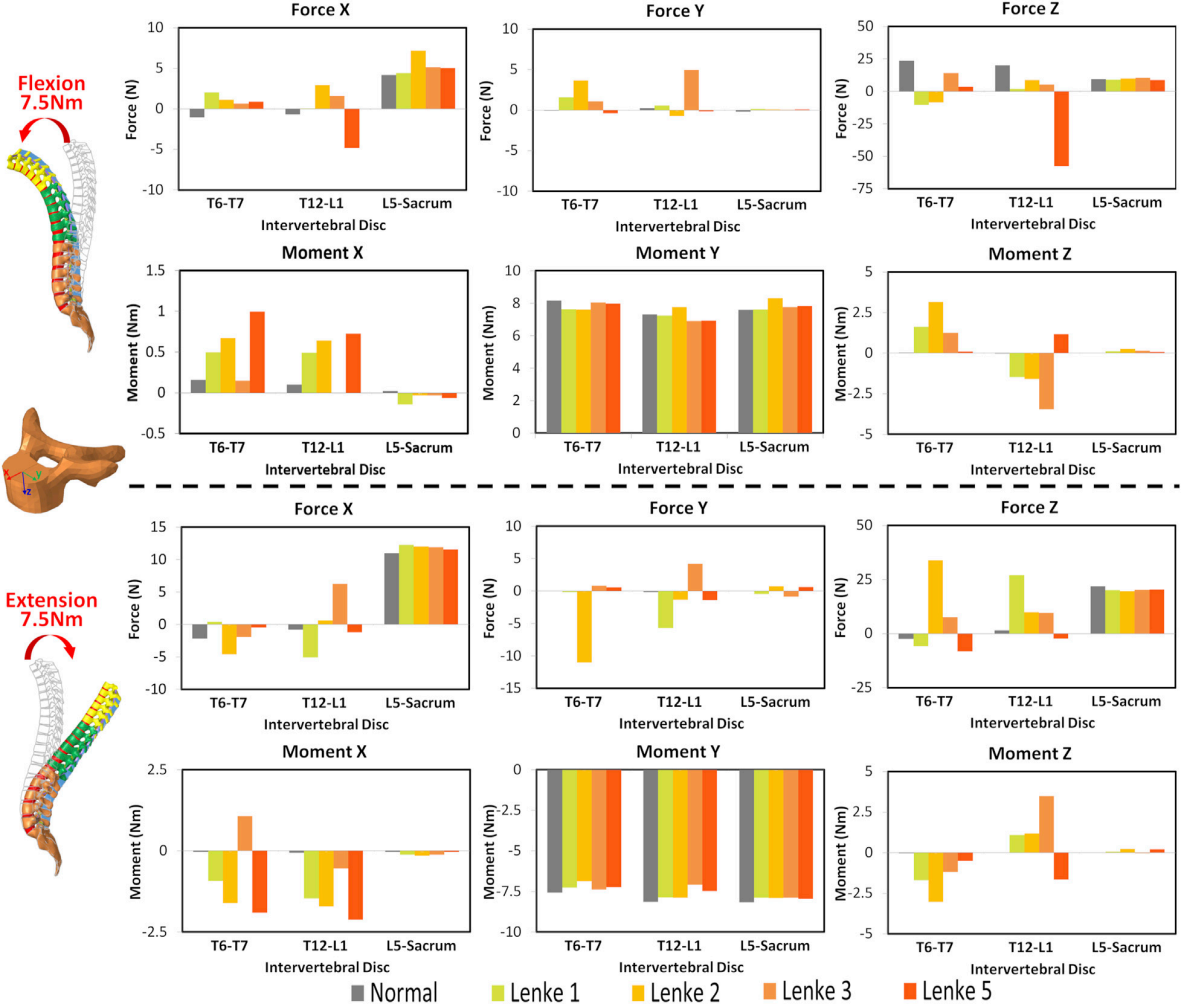
Moments and forces sustained by the IVDs of T6-T7, T12-L1, and L5-Sacrum under flexion-extension loadings for normal and AIS (Lenke 1, Lenke 2, Lenke 3, and Lenke 5) thoracolumbar spine models.

**FIGURE 6:**
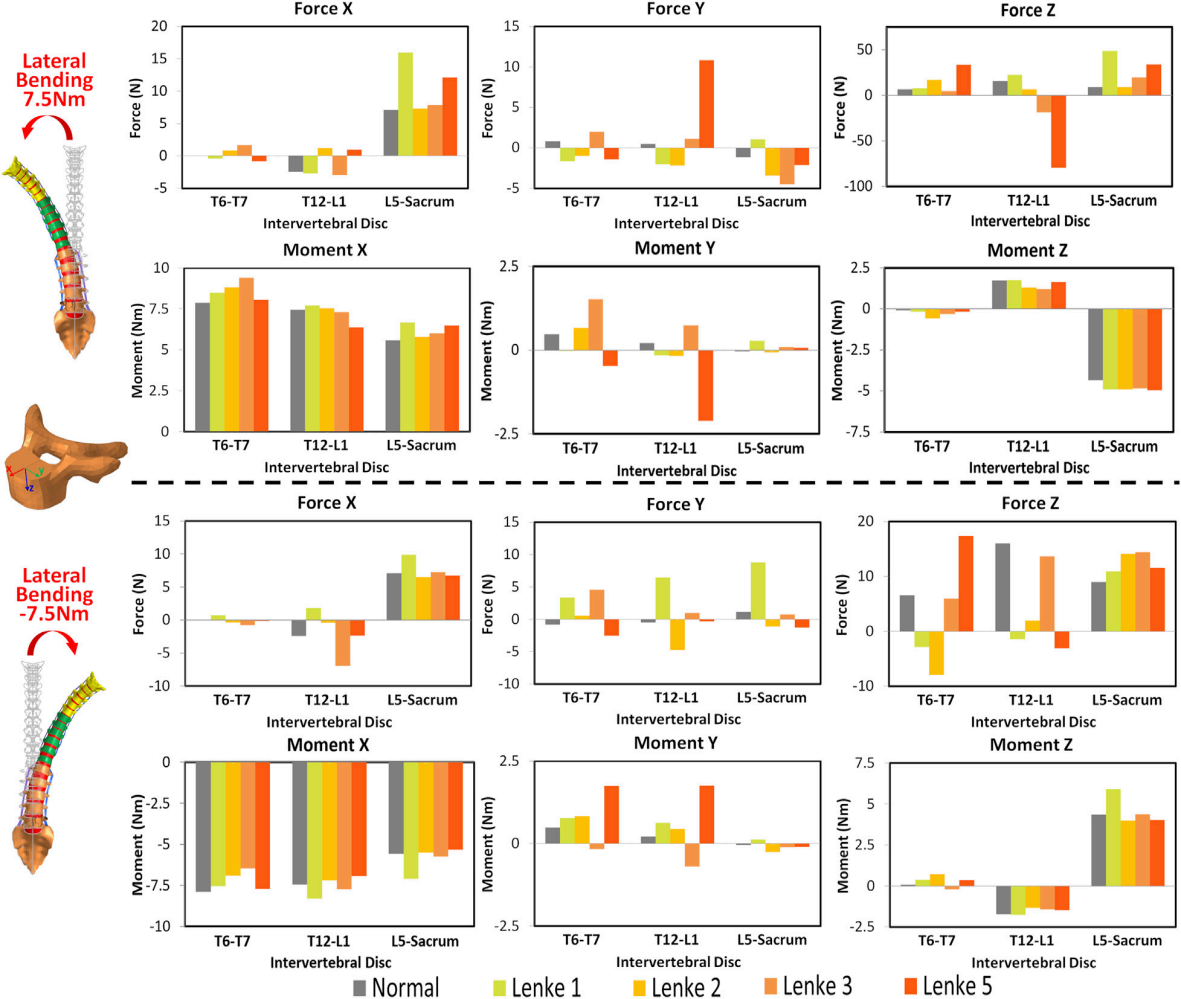
Moments and forces sustained by the IVDs of T6-T7, T12-L1, and L5-Sacrum under lateral bendings for normal and AIS (Lenke 1, Lenke 2, Lenke 3, and Lenke 5) thoracolumbar spine models.

**FIGURE 7:**
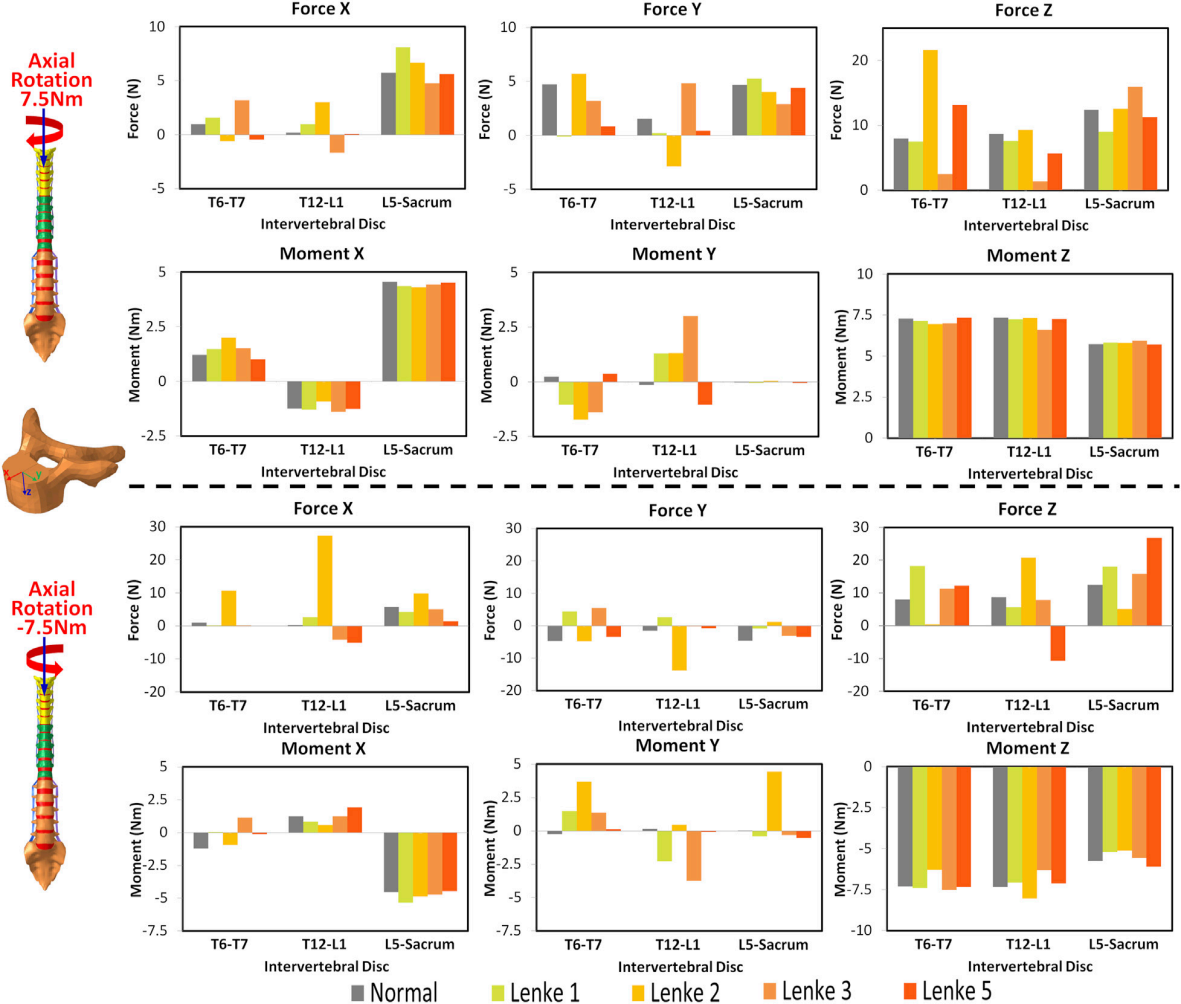
Moments and forces sustained by the IVDs of T6-T7, T12-L1, and L5-Sacrum under axial rotation loadings for normal and AIS (Lenke 1, Lenke 2, Lenke 3, and Lenke 5) thoracolumbar spine models.

The mechanical loadings on the IVDs of the thoracic (T6-T7), thoracolumbar (T12-L1), and lumbosacral (L5-Sacrum) regions were measured and compared among normal and AIS models (Figures 5–7). Under flexion-extension (Figure 5), the primary moments around the Y-axis were close to the applied moments (±7.5Nm) and evenly distributed across all models. However, secondary moments were minimal in the normal model compared to the AIS models, where they were primarily seen in T6-T7 and T12-L1. In the AIS models, Lenke 5 had the highest secondary moments around the X-axis at T6-T7 (1.0Nm during flexion and −1.9Nm during extension) and T12-L1 (0.7Nm during flexion and −2.1Nm during extension). Meanwhile, Lenke 2 and Lenke 3 had the highest secondary moments around the Y-axis at T6-T7 (3.2Nm during flexion and −3.0Nm during extension) and T12-L1 (−3.5Nm during flexion and 3.5Nm during extension), respectively. Both normal and AIS models experienced anterior shear forces (4.2–7.2N along the positive X-axis) and compression forces (7.1–10.4N along the positive Z-axis) during 7.5Nm flexion, with these forces increasing further during 7.5Nm extension.

Under a right lateral bending moment (Figure 6), the principal moments around the X-axis decreased across spinal segments (T6-T7 > T12-L1 > L5-Sacrum) for all models except Lenke 5. This trend continued under a left lateral bending moment of −7.5Nm for the normal and Lenke 5 models, while Lenke 1, 2, and 3 showed the highest moments at T12-L1. Secondary moments around the Z-axis, leading to axial rotations, increased from T6-T7 to T12-L1 to L5-Sacrum for all models. In contrast, secondary moments around the Y-axis, indicating flexion-extension motions, were greater in the IVDs of T6-T7 and T12-L1 than in L5-Sacrum. Lenke 5 had notably high secondary moments, with 2.1Nm at T12-L1 under right bending and 1.75Nm at both T6-T7 and T12-L1 under left bending moment of 7.5Nm, exceeding the secondary moments of other models. Lateral bending consistently resulted in higher anterior shear and compression forces at L5-Sacrum compared to T6-T7 and T12-L1.

Under axial rotation moments (Figure 7), the principal moments around the Z-axis were consistently lower in the IVDs of L5-Sacrum compared to T6-T7 and T12-L1 across all models, while lateral bending (secondary) moments were higher in L5-Sacrum IVDs. Notably, under a right axial rotation moment of 7.5Nm, Lenke 2 exhibited a compression force of 21.6N at T6-T7, significantly higher than that of other models, and a left shear force at T12-L1, unlike the right shear forces in other IVDs. Under a left axial rotation moment of 7.5Nm, the IVD of T12-L1 in Lenke 2 experienced shear forces of 27.3N and 13.8N in the anterior and leftward directions, respectively, also much higher than other models. Meanwhile, the IVD of T12-L1 in Lenke 5 sustained a tension force of 10.6N, contrasting with the compression forces observed in other models.

### 3.3 Intervertebral disc stress analysis

**FIGURE 8:**
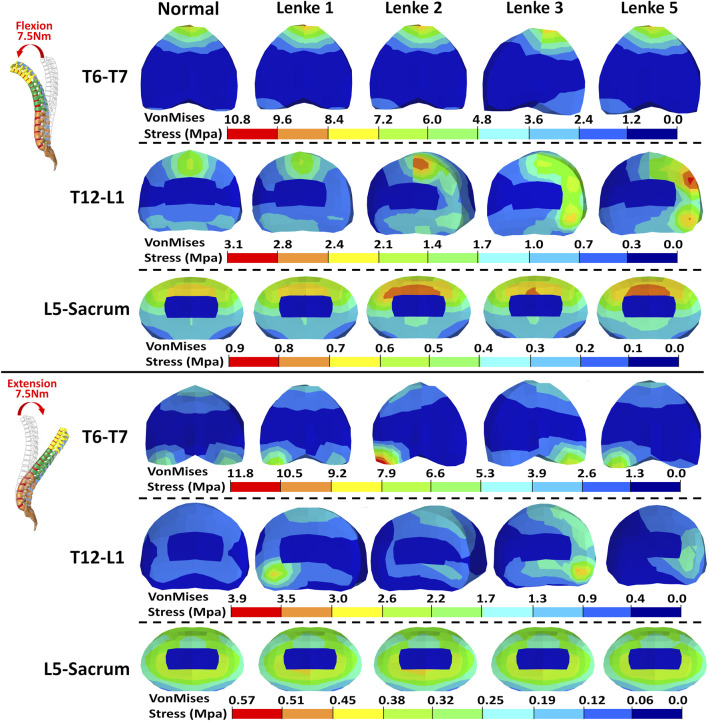
VonMises stress distribution on the IVDs of T6-T7, T12-L1, and L5-Sacrum under flexion-extension loadings for normal and AIS (Lenke 1, Lenke 2, Lenke 3, and Lenke 5) thoracolumbar spine models.

**FIGURE 9:**
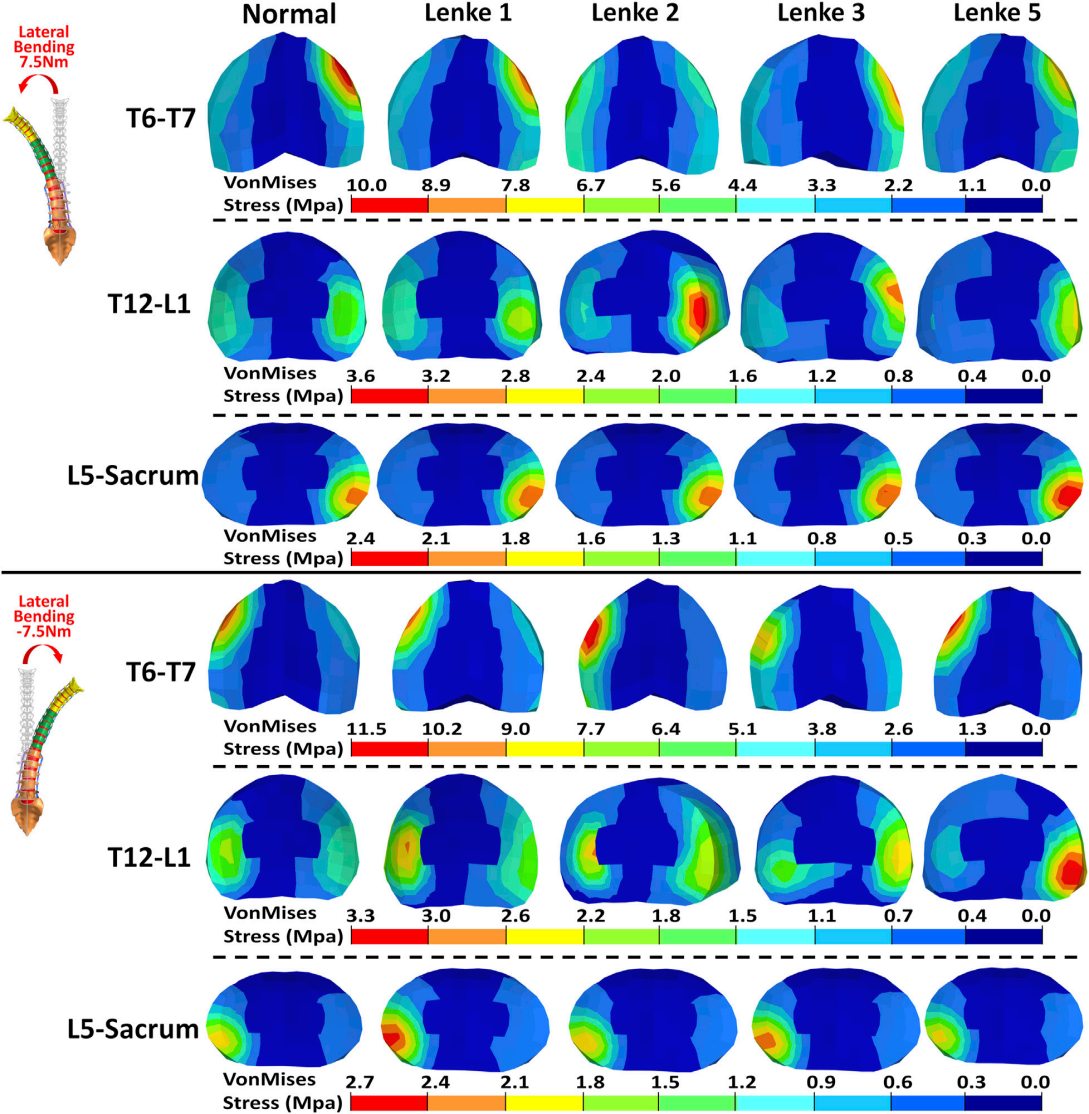
VonMises stress distribution on the IVDs of T6-T7, T12-L1, and L5-Sacrum under lateral bendings for normal and AIS (Lenke 1, Lenke 2, Lenke 3, and Lenke 5) thoracolumbar spine models.

**FIGURE 10:**
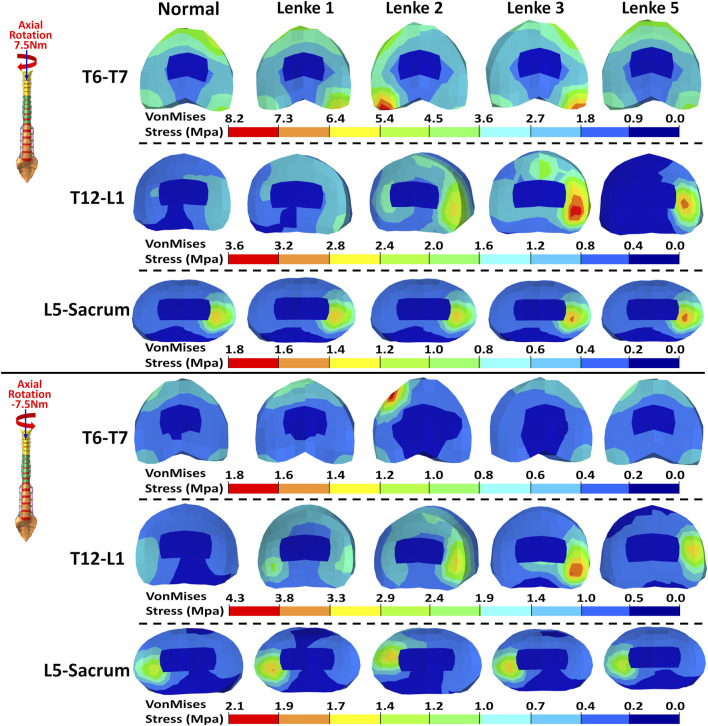
VonMises stress distribution on the IVDs of T6-T7, T12-L1, and L5-Sacrum under axial rotation loadings for normal and AIS (Lenke 1, Lenke 2, Lenke 3, and Lenke 5) thoracolumbar spine models.

**FIGURE 11:**
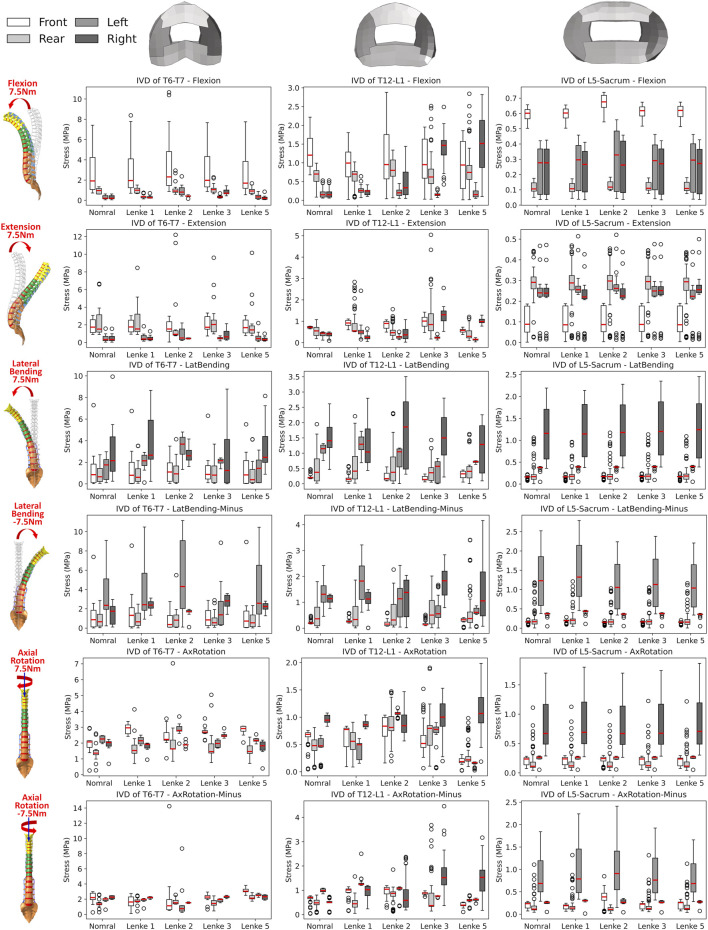
Boxplot of VonMises stress in the front, rear, left, and right regions of the IVDs at T6-T7, T12-L1, and L5-Sacrum under flexion-extension, lateral bending, and axial rotation loadings for normal and AIS (Lenke 1, Lenke 2, Lenke 3, and Lenke 5) thoracolumbar spine models.

The VonMises stress distribution on the IVDs of T6-T7, T12-L1, and L5-Sacrum was examined under flexion-extension (Figure 8), lateral bending (Figure 9), and axial rotation (Figure 10) loadings for normal and AIS (Lenke 1, Lenke 2, Lenke 3, and Lenke 5) thoracolumbar spine models. A more detailed stress distribution for the front, rear, left, and right regions of the IVDs is presented in Figure 11.

Under flexion-extension loadings, the stress distribution patterns on the IVDs of T6-T7 and L5-Sacrum were similar across normal and AIS models (see Figures 8, 11). During 7.5Nm of flexion, the frontal region of the IVDs at T6-T7 and L5-Sacrum exhibited the highest stress loadings, ranging from 7.41MPa to 10.66MPa and from 0.66MPa to 0.74MPa, respectively. The T12-L1 IVD’s frontal region showed peak stress in the normal (2.22MPa), Lenke 1 (1.81MPa), and Lenke 2 (2.88MPa) models, while the rear region peaked in the Lenke 3 (2.51MPa) and Lenke 5 (2.84MPa) models. During 7.5Nm of extension, the rear regions of the IVDs at T6-T7 and T12-L1 consistently had the highest stress loadings, ranging from 6.65MPa to 12.21MPa and from 1.08MPa to 5.04MPa, respectively. The lateral regions of the IVDs at L5-Sacrum also showed high stress, ranging from 6.65MPa to 12.21MPa, across all models.

Under lateral bending, the stress distribution patterns on the IVDs of T6-T7, T12-L1, and L5-Sacrum were similar for both normal and AIS models (see Figures 9, 11). During right lateral bending at 7.5Nm, the right region of most IVDs had the highest stress, with peak levels ranging from 8.14MPa to 9.93MPa at T6-T7, 2.26MPa–3.50MPa at T12-L1, and 2.14MPa–2.45MPa at L5-Sacrum. Notably, the T6-T7 IVD of the Lenke 2 model showed the highest stress of 4.80MPa in the left region. In left lateral bending of −7.5Nm, the left region tends to exhibited the highest stress, with peak levels from 8.85MPa to 11.15MPa at T6-T7, 2.42MPa–3.22MPa at T12-L1, and 2.21MPa–2.79MPa at L5-Sacrum. The T12-L1 IVDs of the Lenke 3 and Lenke 5 models had the highest stresses of 2.83MPa and 4.16MPa, respectively, in the right regions.

Under axial rotation loadings, the stress patterns on the L5-Sacrum IVD were nearly identical across normal and AIS models (see Figures 10, 11). For right axial rotation at 7.5Nm, the right region of the L5-Sacrum IVD showed the highest stress, ranging from 1.70MPa to 1.87MPa. The highest stress in the T6-T7 IVD was found in the front region for the normal (2.94MPa) and Lenke 5 (3.07MPa) models, while other AIS models exhibited peak stress in the rear region (2.38MPa–5.04MPa). The highest stress in the T12-L1 IVD was in the right region for the normal (1.08MPa), Lenke 1 (1.04MPa), and Lenke 5 (1.99MPa) models, while the Lenke 2 (1.47MPa) and Lenke 3 (1.91MPa) models showed peak stress in the rear region. For left axial rotation of −7.5Nm, the left region of the L5-Sacrum IVD had the highest stress (1.66MPa–2.41MPa), and the T6-T7 IVD consistently showed peak stress in the front region (2.75MPa–14.24MPa). The T12-L1 IVD showed the highest stress in the left region for the normal (1.09MPa) and Lenke 1 (2.50MPa) models, while other AIS models showed peak stress in the right region (2.37MPa–4.46MPa).

## 4 Discussion

In this study, we investigated the biomechanical behavior of spinal ROM and IVD mechanical loadings in both normal and AIS models under idealized loading conditions using the finite element (FE) simulation approach. By utilizing the mesh morphing technique based on a normal baseline model, we established Lenke 1, Lenke 2, Lenke 3, and Lenke 5 models to match their representative spinal curvatures. Our findings revealed significant differences between the normal and AIS models, underscoring the complex biomechanical interactions present in scoliotic spines.

Previous studies, as noted by Mehkri et al. (2021), have predominantly focused on ROM of specific spinal regions, often neglecting the global ROM. Our study firstly addresses that gap by evaluating both segmental and global spine ROM, along with coupled motion directions, to provide a more comprehensive understanding of spinal biomechanics in untreated AIS patients compared to healthy controls. This study consistently found higher global spine ROM in AIS models compared to the normal model, particularly in the T6-T12 segment, which corresponds to the greatest spinal deformity angle in Lenke 1, Lenke 2, and Lenke 3 models. For instance, the principal ROM of the normal model accounted for only 84%–90% of that observed in the Lenke 1 model under flexion-extension, lateral bending, and axial rotation loadings (see Table 2). This finding aligns with the observations by Galvis et al. (2016), who included only scoliosis patients with Lenke type 1 curves and found greater mobility, especially in spinal segments directly above and below the curve apex. They suggested that compensatory hypermobility near the curve apex increased ROM. AIS patients have been reported to have taller intervertebral discs relative to vertebral width compared to individuals without spinal deformity (Chen et al., 2017; Wren et al., 2017). A larger disc height-to-vertebral width ratio may be associated with greater lateral bending flexibility, suggesting that AIS patients may have more flexible spines than their peers without scoliosis. In contrast, other researchers, such as Struber et al. (2022), have assumed that AIS curvature induces ROM restriction, expecting scoliotic segments to be more rigid than normal segments due to the alignment and local anatomy of each functional unit of spinal deformity. Eyvazov et al. (2017) also found that more severe curves in Lenke 5 patients significantly reduced spinal mobility. These differing observations highlight the complexity of AIS biomechanics. The AIS models in our study were idealized by applying identical loadings as the normal model, without accounting for characteristic deformities in AIS such as spinal axial rotation and the typical reduction in thoracic kyphosis (sagittal flat back). In clinical scenarios, patients exhibit different muscle forces and activities (Struber et al., 2022), which can result in lower moments and, consequently, reduced ROM compared to our models. Additionally, AIS patients often have more significant deformities in vertebral axial rotation and facet joint anatomy (Lee et al., 2020), which were not fully considered in our current models and may further influence ROM. Therefore, while our findings provide valuable insights, it is essential to consider the variability in individual anatomical and muscular characteristics when interpreting these results.

The AIS models exhibited not only higher global ROM but also pronounced asymmetry in segmental ROM, particularly evident in the lumbar spine segment (T12-Sacrum) during lateral bending and axial rotation loadings. For instance, the T12-Sacrum segment of AIS models displayed a ROM ranging from 11.9° to 18.0° under right axial rotation loading and from 18.2° to 38.9° under left axial rotation loading. This asymmetry in spinal ROM was further coupled with significant secondary motions, contrasting sharply with the negligible coupled motions observed in the normal model. Specifically, the T12-Sacrum segment of AIS models sustained coupled axial rotations ranging from 4.2° to 8.6°, significantly higher than the 0.2° observed in the normal model during flexion-extension loadings. The accentuation of coupled rotational movements at the lumbar level during flexion-extension movements may contribute to the occurrence of rotatory dislocation, a specific complication of scoliosis commonly observed in AIS populations (Goel et al., 2021; Jia et al., 2019). These findings also suggest that AIS patients require higher magnitudes of movement and more substantial coupling rotations to bear the same level of loadings. Moreover, the substantial coupling rotations, particularly evident in the T12-Sacrum segment of AIS models, were associated with markedly higher shear forces in the anterior-posterior and left-right directions at the T12-L1 IVD compared to the normal model (Force X and Y displayed in Figures 5–7). For instance, under extension loading, the T12-L1 IVD in the normal model sustained a shear force of 0.83N to the right, whereas it was 5.1N to the right in the Lenke 1 model and 6.2N to the left in the Lenke 3 model. Under flexion loading, the T12-L1 IVD in the normal model sustained a shear force of 0.68N to the right, compared to 2.94N to the left in the Lenke 2 model and 4.8N to the right in the Lenke 5 model. Therefore, we speculate that the asymmetric structure of AIS patients may first lead to asymmetric spinal motion, then elevate coupled motions, and finally increase IVD loadings, especially in the lumbar segment. These findings may partially explain, from a biomechanical perspective, why AIS patients experience low back pain more often than the normal population, as observed clinically (An et al., 2023). Higher coupled spinal motions outside the sagittal plane have previously been associated with the incidence of low back pain (Cheng et al., 2013), and our findings may complement this understanding. To date, coupled motions and the associated IVD loadings have not been extensively reported in the AIS literature. Our study contributes novel insights by elucidating these complex biomechanical interactions, highlighting the need for further research to better understand their implications for spinal health in AIS patients compared to healthy individuals.

A detailed examination of IVD loadings in terms of VonMises stress in our study indicated that the IVD loading distributions are sensitive to the applied loadings in both normal and AIS models, underscoring the complexity and region-specific nature of the spine’s mechanical environment. The frontal IVD regions of T6-T7, T12-L1, and L5-Sacrum generally exhibited the highest stress under flexion loading, while the stress in these regions was significantly reduced under extension loading. This aligns with previous findings that IVD pressure is greatly reduced in extension loadings for normal lumbar models and those with degenerative IVDs (Park et al., 2013; Zahari et al., 2017). Therefore, the frontal IVD regions may be particularly vulnerable to flexion-induced stress and potential injury. Conversely, the reduced stress during extension loading suggests a relative mechanical unloading, which could be could be therapeutically advantageous. This has clinical implications, as extension-based exercises have been shown to alleviate low back pain (Adams et al., 2000; Park et al., 2024) and support disc healing in certain patients with disc herniation (Oakley and Harrison, 2017).

During right lateral bending, the right regions of these IVDs consistently sustained the highest stress, whereas the left regions experienced the highest stress during left lateral bending. This pattern suggests that lateral bending induces stress on the side toward which the spine bends, indicating a load-bearing asymmetry that could contribute to the progression of scoliosis and associated discomfort. Such insights could influence clinical approaches by reinforcing the importance of targeted therapies or corrective braces that address the asymmetric loading and mitigate further degeneration or pain on the bending side. Interestingly, the IVDs of T6-T7 and T12-L1 demonstrated a relatively even stress distribution under axial rotation loadings, indicating a balanced load-bearing capacity in these areas. In contrast, the right IVD regions of L5-Sacrum predominantly sustained the highest stress under right axial rotation loading, while the left regions bore the highest stress under left axial rotation loading. This localized stress accumulation in the lower lumbar spine may stem from anatomical and biomechanical variations, which should be carefully considered when devising treatment strategies involving rotational exercises or rehabilitation targeting these areas.

A notable exception was observed in the Lenke 3 and Lenke 5 models, where the right IVD regions of T12-L1 consistently sustained higher stress compared to other regions of this IVD, regardless of the applied loadings. This persistent high stress could be attributed to local structural anomalies or specific curvature patterns in these models, leading to uneven load distribution. As shown in Figure 2, the T12-L1 IVDs in Lenke 3 and Lenke 5 models are not at the apex of the structural AIS curve but have a lower IVD height on the right side compared to the left, resulting in coronal wedge angles of 8.0° and 8.5°, respectively. These angles fall within the range (1.5°–9.0°) of T12-L1 IVD coronal wedge angles measured for AIS patients with thoracic and/or lumbar curves (Cheung and Cheung, 2022). The significantly higher stress on the right (concave) T12-L1 IVD regions of the Lenke 3 and Lenke 5 models aligns with the higher mechanical loadings measured on the concave growth plates of AIS patients (Kamal et al., 2019). This elevated stress on the concave IVD regions may increase the risk of IVD degeneration or pain in these specific areas, driven by factors such as higher levels of cell death, increased apoptosis, loss of aggrecan, and increased aggregate modulus (Walter et al., 2011).

The stress distribution patterns identified in this study offer valuable insights for the clinical management of AIS. Therapeutic interventions, such as exercise regimens or bracing, can be designed to counterbalance the stress concentrations observed during flexion-extension, lateral bending, and axial rotation. For instance, exercises that emphasize extension could help relieve stress in the anterior portions of the IVDs, particularly in the lumbar and thoracic regions. Bracing or strengthening exercises that redistribute stress more evenly across the IVDs may help slow the progression of scoliosis and alleviate discomfort caused by asymmetric loading. The elevated stress observed on the concave regions of the T12-L1 IVD in Lenke 3 and Lenke 5 models further highlights the importance of early intervention, such as physical therapy or targeted surgical strategies, to mitigate the risk of IVD degeneration and associated pain. Understanding these specific stress distribution patterns allows for the development of more precise, biomechanically informed treatment plans, potentially improving patient outcomes and quality of life. Moreover, these findings provide a basis for future clinical research to refine therapeutic techniques and enhance the effectiveness of interventions tailored to the unique biomechanics of AIS patients.

## 5 Limitations and future work

This study has several limitations that need to be addressed. Firstly, the AIS models used in our analysis were idealized and did not fully capture the complexity of real-life anatomical variations. Specifically, we did not account for factors such as apical vertebral wedging, specific axial rotation deformities, facet joint anomalies, and the shift of the nucleus toward the convex side, which are commonly observed in AIS patients. These simplifications may lead to an underestimation or overestimation of the biomechanical stresses and motions. Additionally, capturing the unique morphology of each vertebra presents considerable challenges due to the inherent complexity and variability among patients. Therefore, our findings underscore the need for further investigation in future studies to incorporate more realistic vertebral morphology and enhance our understanding of the biomechanical behavior in AIS.

Furthermore, our study primarily focused on the biomechanical aspects of AIS, without considering the long-term effects of these stresses on the progression of scoliosis or the development of associated pathologies such as low back pain and IVD degeneration. Future work should aim to include longitudinal studies to better understand these relationships over time. Integrating patient-specific data into the models could also enhance their accuracy and applicability to clinical practice. Additionally, the potential benefits of various therapeutic interventions, such as specific exercises and bracing techniques, should be evaluated in more detail through clinical trials to confirm their effectiveness in mitigating the identified biomechanical stresses.

## 6 Conclusion

This study analyzed the biomechanical behavior of spinal ROM and IVD mechanical loadings in normal and AIS models under idealized loading conditions using FE simulation. The baseline thoracolumbar FE model was derived from a comprehensive human body FE model initially developed for pedestrian safety in vehicle accidents. This thoracolumbar FE model was validated and calibrated against spinal biomechanical responses under dynamic compression and quasi-static bending conditions, ensuring its accuracy and reliability for the analysis of spinal biomechanics in AIS. By employing the mesh morphing technique on the baseline thoracolumbar FE model, we established AIS models of Lenke 1, Lenke 2, Lenke 3, and Lenke 5 to accurately represent their respective spinal curvatures. Pure bending moments of ±7.5 Nm, aligned with flexion-extension, lateral bending, and axial rotation directions, were applied to both the normal and AIS models. The global spinal ROM, as well as the ROM of spinal segments T1-T6, T7-T12, and L1-Sacrum, were measured for all models under each loading condition. Additionally, the mechanical loadings on the IVDs, including force, moment, and VonMises stress, were evaluated and compared across all models. AIS models consistently exhibited higher principal ROM throughout the thoracolumbar spine segment compared to the normal model, with Lenke 2 displaying the highest ROM, followed by Lenke 3, Lenke 1, and Lenke 5. In the T6-12 spinal segment, which showed the greatest deformity in Lenke 1, Lenke 2, and Lenke 3 models, ROM at the loading direction consistently exceeded that of the normal model, with Lenke 3 displaying the highest ROM. AIS models also showed more pronounced asymmetry in segmental ROM compared to the normal model, especially in the lumbar spine segment (T12-Sacrum) during lateral bending and axial rotation. The mechanical loadings, including moments and forces, sustained by the IVDs in the thoracic (T6-T7), thoracolumbar (T12-L1), and lumbosacral (L5-Sacrum) regions, varied significantly between normal and AIS models. These variations were influenced by the specific spinal curvature types, leading to marked differences in secondary moments and shear forces among the models. For instance, AIS models consistently experienced higher secondary moments and shear forces compared to the normal model, especially under flexion-extension conditions. Under flexion loading, the frontal regions of the IVDs at T6-T7, T12-L1, and L5-Sacrum generally experienced the highest stress, which was notably reduced under extension loading. During right lateral bending, the right regions of these IVDs consistently endured the highest stress, whereas left lateral bending induced the highest stress in the left regions. This observation suggests that lateral bending applies stress on the side towards which the spine bends. The IVDs of T6-T7 and T12-L1 showed even stress distribution under axial rotation, indicating balanced load-bearing capacity. In contrast, the right IVD regions of L5-Sacrum sustained the highest stress under right axial rotation, while the left regions bore the highest stress under left axial rotation. Specifically, in Lenke 3 and Lenke 5 models, the right (concave) regions of the T12-L1 IVD consistently sustained higher stress levels compared to other regions of the same IVD, regardless of the loading conditions applied. Our findings underscore significant biomechanical differences between normal and AIS models, shedding light on the intricate interactions within scoliotic spines. These insights enhance our understanding of AIS biomechanics, offering crucial insights for improved diagnosis, treatment planning, and prognosis. For instance, therapeutic exercises focused on extension may mitigate stress on anterior IVDs, potentially reducing the risk of low back pain or disc herniation, while rotational exercises require careful management to minimize stress in the lower lumbar regions. This study provides foundational insights into the complex biomechanics of AIS and normal spine models under diverse loading conditions, paving the way for future research to refine biomechanical models, explore long-term implications, and devise effective treatment strategies for AIS patients.

## Data Availability

The data that support the findings of this study are available on request from the corresponding authors.
