# Supplementary material for: Biomechanical analysis of spinal range of motion and intervertebral disc loadings in normal and adolescent idiopathic scoliosis models
Source: Front Bioeng Biotechnol. 2025 Feb 19;13:1473776. doi: 10.3389/fbioe.2025.1473776 (PMC11880291; doi:10.3389/fbioe.2025.1473776)
Supplement: Supplementary file 1 [file DataSheet1.docx]

Appendix

# Biomechanical assessment of the thoracolumbar spine FE model

Biomechanical assessment of the thoracolumbar spine FE model encompassed two primary types of loadings: dynamic compression and quasi-static bending (as illustrated in Figure 1 in the manuscript). The dynamic compression assessment was carried out on thoracic spinal segments T5-T7, T8-T10, and T11-L1, with the entire lumbar spine T12-L5 undergoing the same evaluation, employing compression speeds of 1 m/s and 0.1 m/s, respectively. These procedures aligned with established experimental testing protocols [1,2]. Specifically, the individual segmental FE models for T5-T7, T8-T10, T11-L1, and T12-L5 were extracted from the thoracolumbar spine FE model, retaining the IVDs and ligaments within the segments, mirroring the conditions of the corresponding target experiments [1,2]. In this assessment, the lower portions of the inferior vertebrae were constrained across all degrees of freedom, while the upper portions of the superior vertebrae received a prescribed compression load of 12mm. Speeds of 1 m/s were applied for segments T5-T7, T8-T10, and T11-L1, while a speed of 0.1 m/s was used for T12-L5 (refer to Figure 1 in the manuscript). The compression force was gauged at the inferior vertebrae, mirroring the methodology of experimental protocols [1,2]. The compression force-displacement curves generated by the simulations were subsequently compared with corresponding experimental measurements for model evaluations [1,2].

The quasi-static bending assessment encompassed the entire thoracic spine T1-T12 and lumbar spine L1-L5, subjected to pure moments of flexion-extension, lateral bending, and axial rotation. This evaluation was conducted in alignment with established experimental testing protocols [3,4]. Similar to the dynamic compression assessment, the thoracic spine T1-T12 and lumbar spine L1-L5 were isolated from the thoracolumbar FE model while retaining the soft tissue components. Throughout this assessment, the lower portions of the inferior vertebrae (T12 within T1-T12 and L5 within L1-L5) were constrained across all degrees of freedom. The upper portions of the superior vertebrae (T1 within T1-T12 and L1 within L1-L5) were subjected to pure moments corresponding to flexion-extension, lateral bending, and axial rotation directions. The thoracic spine T1-T12 was subjected to pure moments up to ±2.0 Nm, with a progressive rate of 1.0 Nm/s. The lumbar spine L1-L5 was exposed to pure moments up to ±3.75 Nm and ±7.5 Nm, with increasing rates of 2.0 Nm/s. The simulations continued until the models reached a state of stability, characterized by no alteration in load-bearing. The ultimate relative rotations between T1 and T12, as well as L1 and L5, along the principal loading directions of flexion-extension, lateral bending, and axial rotation were gauged to determine the ROM within the simulations. These measurements followed the methodology outlined in the targeted experimental protocols and were subsequently compared with the corresponding experimental measurements to evaluate the model [3,4].

When comparing the model responses with the experimental measurements under dynamic compression loadings and quasi-static bending conditions, the compression force-displacement responses and ROMs measured in the thoracic spinal segments were generally observed to surpass the upper limits of the experimental data, while the results measured in the lumbar spine closely aligned with the experimental data (Figures 1A and 2A). This discrepancy suggested a potential overestimation of stiffness in the thoracic spinal segments, prompting the need for a reduction in stiffness to better match the experimental measurements. To address this, adjustments were made to the material properties of the vertebral components. Specifically, the Poisson’s Ratio of the vertebral spongy bone was modified from 0.45 to 0.25, and a failure threshold of 0.06 for plastic strain was incorporated into the material model of the vertebral spongy bone, following established data from previous studies [2]. Additionally, a failure threshold of 0.071 for the effective plastic strain was also introduced into the material model of the vertebral cortical bone, aligning with reported data from the same source [2]. These adjustments in the material properties of the spinal vertebrae (Table 1 in the manuscript) served the purpose of model calibration, resulting in compression force-displacement responses and ROMs in the thoracic and lumbar spines that more closely matched the experimental data (Figures 1A and 2A).


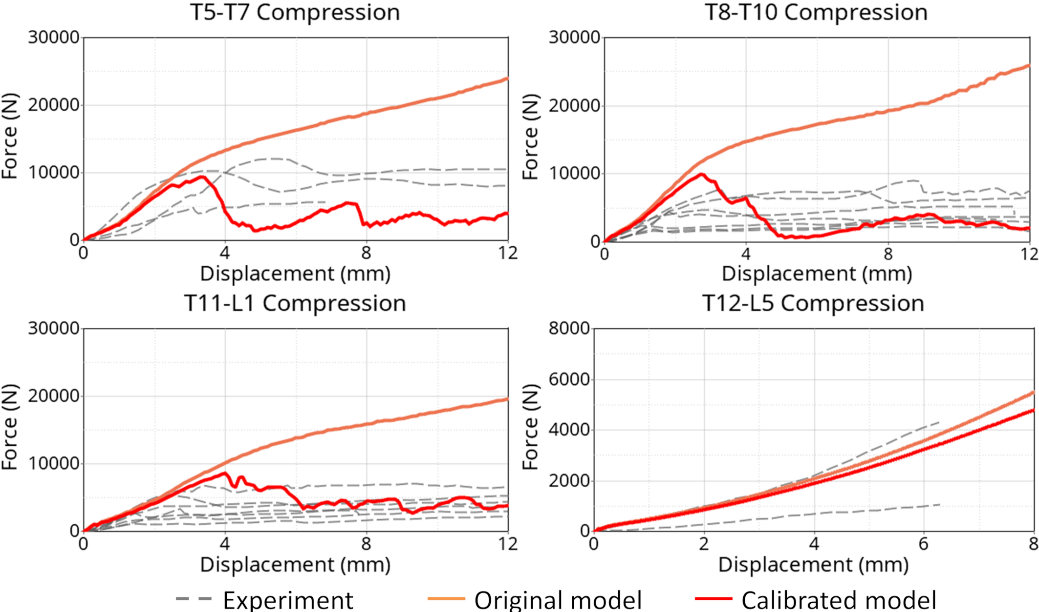


**Figure 1A.** Thoracolumbar spine FE model evaluation and calibration under dynamic compression loadings: experimental measurements of thoracic spinal segments (T5-T7, T8-T10 and T11-L1) come from the study by Wagnac et al. [1] with the measurements of the lumbar spine (T12-L5) from the study by Wagnac et al. [2].


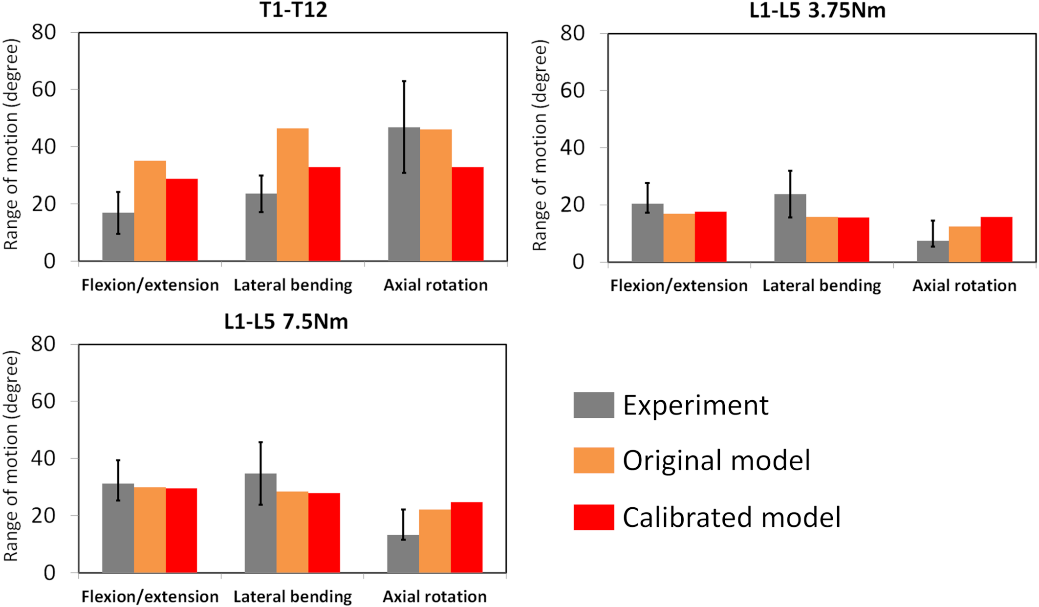


**Figure 2A.** Thoracolumbar spine FE model evaluation and calibration under quasi-static bending conditions: experimental measurements of thoracic spine (T1-T12) come from the study by Liebsch et al. [3] with the measurements of the lumbar spine (L1-L5) from the study by Rohlmann et al. [4].

# Final thoracolumbar curvatures in pure bending conditions


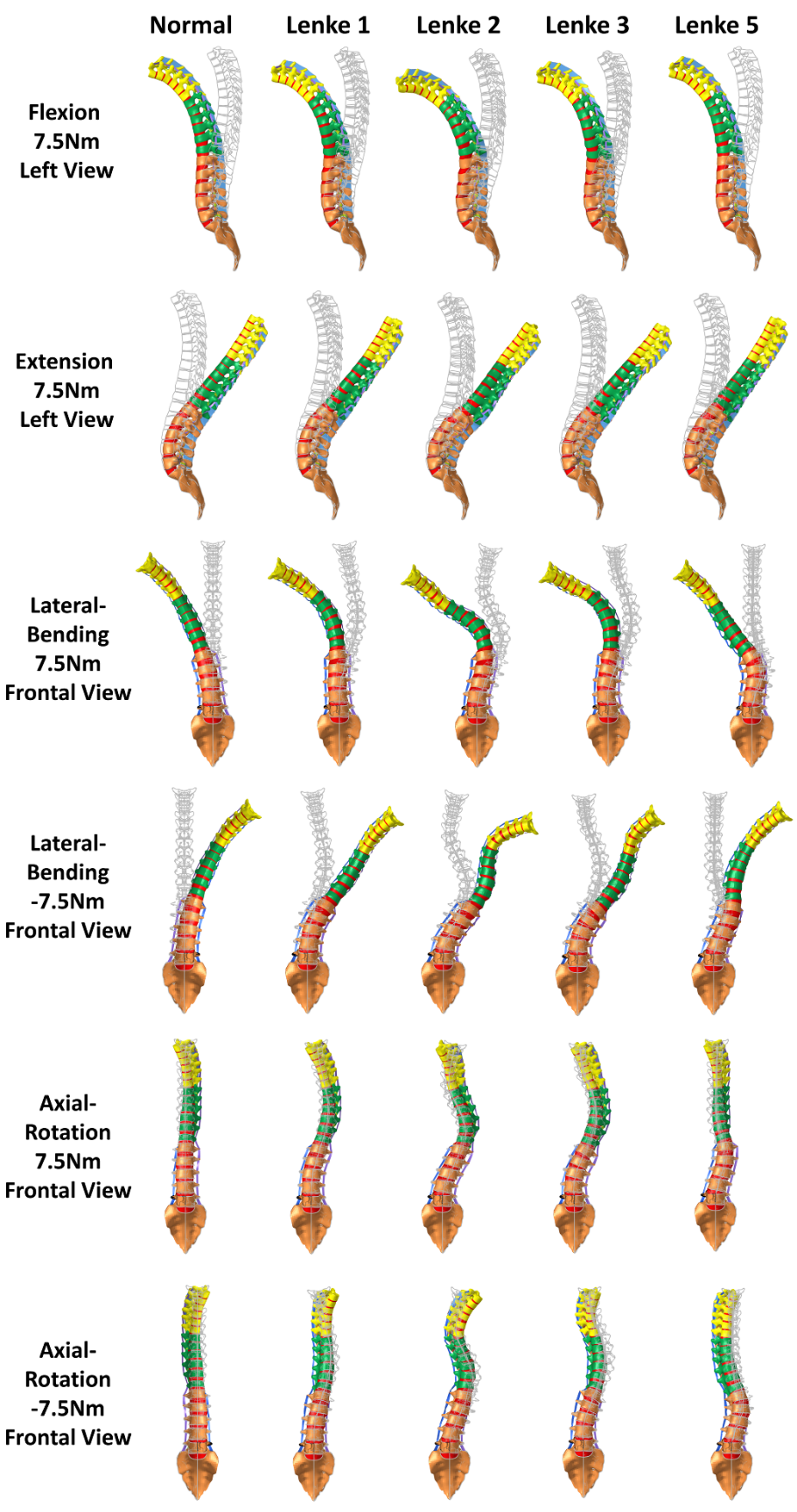


**Figure 3A.** Final thoracolumbar curvatures under ±7.5Nm moments of flexion-extension, lateral bending, and axial rotation in normal and AIS models (Lenke Types 1, 2, 3, and 5). Transparent thoracolumbar curvatures indicate the initial curvatures before applying the loadings.

# References

[1] E. Wagnac, C.-É. Aubin, K. Chaumoître, J.-M. Mac-Thiong, A.-L. Ménard, Y. Petit, A. Garo, P.-J. Arnoux, Substantial vertebral body osteophytes protect against severe vertebral fractures in compression, Plos One 12 (2017) e0186779. https://doi.org/10.1371/journal.pone.0186779.

[2] E. Wagnac, P.-J. Arnoux, A. Garo, C.-E. Aubin, Finite element analysis of the influence of loading rate on a model of the full lumbar spine under dynamic loading conditions, Medical & Biological Engineering & Computing 50 (2012) 903–915. https://doi.org/10.1007/s11517-012-0908-6.

[3] C. Liebsch, N. Graf, K. Appelt, H.-J. Wilke, The rib cage stabilizes the human thoracic spine: An in vitro study using stepwise reduction of rib cage structures, PloS One 12 (2017) e0178733. https://doi.org/10.1371/journal.pone.0178733

[4] A. Rohlmann, S. Neller, L. Claes, G. Bergmann, H.-J. Wilke, Influence of a follower load on intradiscal pressure and intersegmental rotation of the lumbar spine, Spine 26 (2001) E557–E561. https://doi.org/10.1097/00007632-200112150-00014.
